# Influenza vaccination coverage in children with inflammatory bowel disease

**DOI:** 10.1111/irv.12236

**Published:** 2014-01-03

**Authors:** Aleksandra Banaszkiewicz, Beata Klincewicz, Izabella Łazowska-Przeorek, Urszula Grzybowska-Chlebowczyk, Paulina Kąkol, Aleksandra Mytyk, Anna Kofla, Andrzej Radzikowski

**Affiliations:** a Department of Pediatric Gastroenterology and Nutrition, Medical University of WarsawWarsaw, Poland; b Department of Pediatric Gastroenterology and Metabolic Diseases, Poznan University of Medical SciencesPoznan, Poland; c Gastroenterology Division, Department of Pediatrics, Medical University of SilesiaSilesia, Poland; d Student Research Association, Medical University of WarsawWarsaw, Poland; e Department of Pediatrics, Gastroenterology and Nutrition, Medical University of WroclawWroclaw, Poland

**Keywords:** Crohn's disease, Immunocompromised, Influenza, ulcerative colitis, vaccine

## Abstract

The aim of this study was to evaluate the influenza vaccination status among paediatric patients with inflammatory bowel disease (IBD) in Poland. This was a questionnaire-based study. 242 patients with IBD and 142 controls were enrolled in the study. Of patients with IBD, 7·8% received an influenza vaccine, compared to 18·3% of controls (*P* = 0·0013). There were no statistically significant differences in time from IBD diagnosis, disease activity and in drugs, between vaccinated and non-vaccinated IBD children. In conclusion, the data of our study demonstrate an alarmingly poor influenza vaccination status in the majority of children with IBD. Therefore, there is an unmet need to implement better influenza vaccination strategies for this group of patients.

## Introduction

Inflammatory bowel diseases (IBD), including ulcerative colitis (UC) and Crohn's disease (CD), are multifactorial polygenetic diseases with genetic heterogeneity. It is hypothesised that IBD is a result of an impaired or altered immune response to environmental and/or infectious factors.^1^ IBD predominantly affects the gastrointestinal system, but it is also associated with many extra-intestinal manifestations, including pulmonary disorders, such as bronchiectasis and pulmonary function abnormalities. Long-term treatment for IBD involves the use of anti-inflammatory agents and immunosuppressive medications including steroids, antimetabolites and biological therapies. IBD is usually more complicated in children than in adults. Therefore, the majority of children with IBD are treated more aggressively using a combination of immunosuppressive and biological drugs.

Because of an underlying disease, malnutrition, surgery or immunosuppressive therapy, patients with IBD may be at risk of developing infections, such as influenza.^2^ Additionally, children and adolescents with IBD, similar to the general population, are exposed to well-known risk factors for contracting the influenza virus, which include spending time in school and in day-care centres. Therefore, protecting this group against infections is of particular importance.

A committee formed by the Crohn's and Colitis Foundation of America in 2004 and by the European Crohn's and Colitis Organization stated that patients with IBD would benefit from immunisation for vaccine-preventable diseases and recommended the use of inactivated vaccines for this group.^3^,^4^ According to these recommendations, the most important vaccines for patients with IBD to receive are those against pneumococcal disease and influenza. In Poland, only inactivated vaccines against influenza are registered. Therefore, every patient with IBD, regardless of his/her immunological status, is recommended to receive vaccinations. It has been shown that the inactivated influenza vaccine is immunogenic and safe for use in children with IBD.^5^,^6^ Until now, only a few studies assessing the rate of influenza vaccination have been conducted in patients with IBD. However, none of these studies had a control arm, and all but one study was performed in adult patients.^7^–^9^ The only paediatric study in patients with IBD was an Australian audit that reviewed medical records.^10^ In Australia, the annual influenza immunisation is funded for special risk groups, such as children and adolescents with IBD who are ≥6 months of age. In Poland, the influenza vaccine is strongly recommended. However, the cost of the vaccination is not reimbursed.

The aim of this study was to evaluate the influenza vaccination status among paediatric patients with IBD in Poland.

## Methods

This prospective study was conducted in four university-affiliated hospitals for children in Poland (cities of Warsaw, Wroclaw, Katowice and Poznan) between April and June 2013.

The study group was parents of 2- to 18-year-old children diagnosed with IBD, at different stages of disease and treatment process. Diagnosis of CD or UC was based on clinical signs and symptoms, as well as on endoscopical, histological and radiological results, according to the Porto criteria.^11^ The control group consisted of parents of 2- to 18-year-old patients with functional disorders of the digestive tract without any chronic treatment.

Parents of all the children were asked to fill out a two-part, one-page questionnaire. The first part of the questionnaire consisted of questions regarding age, sex, diagnosis and current treatment (the last two questions only for children with IBD). In the second part of the questionnaire, the parents were asked their opinion about a list of potential arguments for and against influenza vaccination.

The odds ratio and its 95% confidence interval were used as a measure of effect size. The confidence intervals for the differences between the two independent binomial proportions were estimated using the Agresti–Caffo method. The confidence interval for the difference between two dependent proportions was performed using the Wald procedure with the Agresti and Min modification. McNemar's test was used for two dependent proportions. A global test for the difference between two sets of dependent proportions was estimated with 9999 bootstrapped samples. The chi-square test for two proportions and an exact test, if necessary, were used for cross-classification tables. The median was used as location parameter, and its statistic was computed as the measure of variability.^12^ Confidence intervals for the difference between two medians were estimated using the studentised bootstrap approach.

## Results

In total, 242 patients and 142 controls were enrolled in this study. None of the patients enrolled refused to participate. The baseline characteristics for the patients and their influenza vaccination rates are shown in Table1. There was no difference in the vaccination rate between CD and UC patients (*P* = 0·812). Children with IBD were two times less likely to be vaccinated against influenza, compared to the controls (OR=2·6, 95% CI: 1·4–4·9). There were no statistically significant differences in the time from IBD diagnosis, disease activity or IBD therapy between vaccinated and non-vaccinated IBD children, which is presented in Table2.

**Table 1:** Baseline characteristics of study groups

Group	*N*	Season 2012/13			Sex			Age (years)	
		
		Vaccinated	Non-vaccinated	%	M	F	% M	Med	S_n_
Control	142	26	116	18·31 95% CI (12·3; 25·7)	73	69	51·41	10	4
CD*	126	9	117	7·14 95% CI (3·3; 13·1)	71	55	56·35	14	3
UC	116	10	106	8·62 95% CI (4·2; 15·3)	56	60	48·28	14	3
IBD	242	19	223	7·85 95% CI (4·8; 12·0)	127	115	52·48	14	3
Control – IBD		10·46% *95% CI (3·3%; 17·9%)* *P* = 0·0013			−1·07% *95% CI (−11·4%; 9·2%)* *P* = 0·8385			*−*4 years 95% CI (*−*5; *−*3) *P* < 0·0001	

CD, Crohn's disease; IBD, inflammatory bowel disease; F, female; UC, ulcerative colitis; M, male; Med, median; *N*, number; S_n_, average dispersion.

* No differences between CD and UC groups.

**Table 2:** IBD patients' characteristics

Vaccinated in 2012/13		Remission	IBD flare	Mesalazine	Azathioprine	Infliximab/adalimumab	Time from IBD diagnosis
Yes	Yes	15	3	18	9	1	Med = 3S_n_ = 2
	No	4	16	1	10	18	
	% Yes	78·95	15·79	94·74	47·37	5·26	
No	Yes	144	79	181	119	26	Med = 2S_n_ = 1·5
	No	79	144	42	104	197	
	% Yes	64·57	35·43	81·17	53·36	11·66	
		OR = 2·1 95% CI (0·7; 6·4) *P* = 0·3136	OR = 0·34 95% CI (0·1; 1·2) *P* = 1275	OR = 4·2 95% CI (0·5; 32) *P* = 0·2109	OR = 0·8 95% CI (0·3; 2·1) *P* = 0·64	OR = 0·42 95% CI (0·05; 3·3) *P* = 0·704	Diff = 1 95% CI (*−*0·9; 1·3) *P* = 0·5244

IBD, inflammatory bowel disease; Med, median; S_n_, average dispersion.

Among the reasons parents chose to vaccinate their children were their belief in influenza vaccine efficacy, fear of influenza complications, as well as their doctor's advice; there were no statistically significant differences between IBD and controls in that respect (Table3). The parents' most common reasons for not vaccinating their children with IBD included fear of influenza vaccination side effects and lack of belief in influenza vaccine efficacy. In controls, the reasons were lack of belief in influenza vaccine efficacy and doctor's advice against vaccination. Differences between the two groups in this respect were statistically significant (Table4).

**Table 3:** Reasons for receiving influenza vaccination

Group	IBD			Controls			OR	95% CI
		
Reasons	Yes	No	%Yes	Yes	No	%Yes		
I am afraid of influenza complications	17	2	89·47	21	5	80·77	2·02	0·3; 11·8
I believe in influenza vaccine efficacy	18	1	94·74	23	3	88·46	2·35	0·2; 24·5
My doctor recommends influenza vaccine	15	4	78·95	22	4	84·62	0·68	0·1; 3·2
	*P* = 0·6999							

IBD, inflammatory bowel disease.

**Table 4:** Reasons for not receiving influenza vaccination

	CD			UC			IBD (CD + UC)			Controls			OR*	95% CI

	Yes	No	% Yes	Yes	No	% Yes	Yes	No	% Yes	Yes	No	% Yes		
I am afraid of influenza vaccine side effects	67	50	57·26	58	48	54·72	125	98	56·05	47	69	40·52	1·87	1·19; 2·95
I do not believe in influenza vaccine efficacy	38	79	32·48	47	59	44·34	85	138	38·12	60	56	51·72	0·58	0·37; 0·91
My doctor advises against influenza vaccine	46	71	39·32	36	70	33·96	82	141	36·77	50	66	43·1	0·77	0·49; 1·21
I do not have money for influenza vaccine	8	109	6·84	10	96	9·43	18	205	8·07	11	105	9·48	0·84	0·38; 1·84
	*P* = 0·3351						*P* = 0·0076							

CD, Crohn's disease; UC, ulcerative colitis; IBD, inflammatory bowel disease.

* IBD/controls.

## Discussion

The results of this prospective trial indicate that the influenza vaccination rate is very low in Polish children and adolescents with IBD.

All previously published studies assessing the seasonal influenza vaccination coverage in patients with IBD emphasised that the rates of influenza vaccination were low and varied from 19%^13^ to 28% in adults^7^,^14^ and that this rate was only 10% in children.^10^ However, the vaccination rate in our study was <8%. Several factors could explain the results observed in our study. First, many of the recommended vaccines, including the influenza vaccine, are not reimbursed in Poland. The high price of the influenza vaccine (approximately 10 Euros) may account for the low vaccination rate. However, the influenza vaccine is one of the cheapest vaccines available in Poland. Second, the vaccination schedule is divided into two parts: the first part is funded and is called ‘obligatory vaccines’, while the other part is not funded and is called ‘recommended vaccines’. The label of ‘recommended vaccines’ could incorrectly suggest that these vaccines are voluntary or less important. Third, many doctors and nurses do not emphasise the necessity of vaccination against influenza and, alternatively, recommend vaccination against invasive pneumococcal or meningococcal infections. Finally, many individuals advocate against vaccinations in general, which could influence the public's attitudes. Moreover, in Poland, the influenza vaccination coverage is low and has been continuously decreasing over the last several years. During the influenza season in 2012/2013, only 3·7% of Poles were vaccinated against influenza, compared to prior seasons (4·5% in 2011/2012 and 5·2% in 2010/2011). In part, this could be the result of a decision by the Polish Ministry of Health not to purchase the H1N1 vaccine in 2010, which could have implied that it was not important to receive the influenza vaccine. Taken together, these factors likely impacted the vaccination status in children with IBD. However, it should be noted that the rate of influenza vaccinations in healthy children is low as well.

We did not observe a correlation between vaccination rate and either IBD treatment or IBD activity. However, it was previously demonstrated by Narula *et al*. 8 that immunosuppression was associated with a decreased uptake of influenza vaccines in Canadian adults. In Canada, there are two types of vaccines against influenza: a live, attenuated, intranasal vaccine, which is not recommended for immunosuppressed patients, and an inactivated vaccine, which is recommended for such patients. In Poland, only inactivated vaccines are available. Therefore, patients with IBD do not need to be concerned about which vaccine to receive. This finding could explain why there was no difference in vaccination rates between immunosuppressed and non-immunosuppressed IBD children.

Children with IBD were more than twice as likely to not be vaccinated, compared to the controls. Because we included a control group in our study, we observed that patients with IBD are vaccinated less than healthy people, while in all prior studies, the results were only compared to data from the general population. The use of data from the general population rather than a control group could increase the range of the data. For example, the general influenza vaccination rate in Poland was 4%, while in our study, the vaccination rate was 18%.

In the present study, the majority of parents of both patients and controls decided not to vaccinate their children against influenza. We found that most parents of IBD children ‘were afraid of the vaccine's side effects’. It could be that they do not realise that influenza vaccine is very safe in patients with IBD. Most parents of controls said that they ‘did not believe in vaccine efficacy’. The explanation of this lack of confidence may be some people's belief that influenza vaccine will protect them against all flu-like diseases. Consequently, if they contract any respiratory infection after having been vaccinated, they are disappointed. It appears that there is a lack of reliable information about both the influenza vaccine itself and its indisputable benefits, especially for this group of patients, which accounts for many individuals' decisions to not to vaccine their children. Therefore, there is a strong need to educate patients and their families better in order to improve influenza vaccination rates.

The most commonly cited arguments for immunisation were as follows: belief in vaccine efficacy, fear of side effects from the vaccine and lack of a doctor's recommendation. These arguments were chosen with similar frequency by patients and by the control group. Therefore, it appears that, if parents were educated on the need for vaccination, they would vaccinate their children regardless of the child's health conditions.

The strengths of our study include the use of several centres and the use of a control group, which enabled us to obtain the true immunisation rate among patients with IBD. For the first time, we demonstrated that there was no difference in the rate of vaccination between CD and UC patients. No prior studies evaluating vaccination coverage performed this type of subanalysis. The limitation of our study is the limited number of close-end answers in the questionnaire.

In conclusion, the data from our study demonstrate an alarmingly low influenza vaccination rate in the majority of children with IBD. Therefore, there is an unmet need to implement better influenza vaccination strategies for this group of patients. The surveillance of patients' vaccination schedule needs to be an integral part of IBD care.
